# Barriers to patient and family‐centred care in adult intensive care units: A systematic review

**DOI:** 10.1002/nop2.253

**Published:** 2019-03-28

**Authors:** Frank Kiwanuka, Shah Jahan Shayan, Agbele Alaba Tolulope

**Affiliations:** ^1^ International Campus Tehran University of Medical Sciences Tehran Iran; ^2^ School of Nursing Kabul University of Medical Sciences Kabol Afghanistan; ^3^ Department of Medical Physics, School of Medicine Tehran University of Medical Sciences Tehran, Iran; ^4^ Department of Basic Medical Sciences College of Health Sciences and Technology Ijero‐Ekiti Nigeria

**Keywords:** adult critical care unit, barriers, critical care, critical care nursing, healthcare providers, intensive care unit, nursing, patient and family‐centred care, physicians, systematic review

## Abstract

**Aim:**

Despite remarkable theoretical evidence of positive outcomes of patient and family‐centred care, it is rarely performed in the intensive care setting. The aim of this review was to assess the barriers to patient and family‐centred care among healthcare providers, patients and family members in adult intensive care units.

**Design:**

A systematic review of both qualitative and quantitative studies.

**Methods:**

The search strategy sought for published peer‐reviewed research papers limited to English language from conception to 2018. The review protocol was registered in the CRD Prospero database (CRD42018086838). Literature search was carried out in four databases: EMBASE, Cochrane Library, PubMed and Scopus where keywords “barriers,” “patient and family centered care,” “patient‐centered care” and “intensive care unit” appeared in any part of the reference. Hand search of reference lists of identified papers was also done to capture all pertinent materials. Each study was assessed by three independent reviewers against the inclusion criteria. Evidence was graded according to sampling quality, quantity and measurement of intended outcomes. Screening of studies and citations resulted in seven studies that were included in the analysis.

**Results:**

Barriers to patient and family‐centred care broadly fall under four categories; lack of understanding of what is needed to achieve patient and family‐centred care, organizational barriers, individual barriers and interdisciplinary barriers.

## 1 INTRODUCTION

The population of critically ill patients and survival from critical illnesses are increasing with advancement in intensive care approaches (Maguire & Carson, 2013). Although advances in medical science have provided new options and improved prognosis, consequently they have distanced healthcare providers (HCP) from their patients (Barry & Edgman‐Levitan, 2012). This has created a healthcare environment where patients and families are sidelined from crucial discussions and oftentimes left in the grey zone without information on treatment options, diagnostics and how their problems are being managed (Barry & Edgman‐Levitan, 2012).

Attempts to change disease‐focused care have mainly considered and advocated for the patient and family‐centred care (PFCC) approach. The Institute of Medicine (IOM) earmarked six dimensions of healthcare improvement which included the following (a) patient‐centred care; (b) care should be respectful of patients’ values, preferences and expressed needs; (c) coordinated and integrated; (d) involve provision of information, communication and education; (e) ensure physical comfort; and (f) care that provides emotional support, relieves fear and anxiety and care that involves family and friends (Tzelepis et al., 2014). Indeed, the IOM report increased the enthusiasm of implementing PFCC in hospitals (Ciufo, Hader, & Holly, 2011). PFCC has been reported to be successful in achieving the triple aims of healthcare reform identified by IOM including improving patient and family experience of care including quality and satisfaction; improving health outcomes; and reduction in the per capita costs of health care. Noteworthy, patient‐centred intensive care units (ICUs) have been appreciated by professional organizations of both critical care nurses and physicians as the fitting model for patient care (Goldfarb et al., 2017; Kogan et al.,^2016^; Mitchell et al., 2016; Riley, White, Graham, & Alexandrov, 2014).

Although talks about PFCC have gained momentum globally, the greatest challenge has been involvement of patients and families in different dimensions of PFCC (Barry & Edgman‐Levitan, 2012). Barriers to achievement of PFCC across studies include organizational challenges, communication challenges, lack of team coordination and negative perceptions towards PFCC (Tunlind, Granstrom & Engstrom, 2015; Esmaeili, Cheraghi, & Salsali, 2014; Ganz & Yoffe, 2012; Turnbull, Davis, Needham, White, & Eakin, 2016; Wilkes, Marcin, Ritter, & Pruitt, 2016).

For successful achievement of PFCC, clinicians, policymakers and other stakeholders need to acknowledge the barriers associated with its realization. Awareness of barriers to PFCC may be helpful in identifying strategies needed for its successful implementation and sustainability. This formed the basis of this review.

### 1.1 Purpose of the review

Positive outcomes of patient and family‐centred care has become ubiquitous in healthcare globally; however, there are challenges of turning the rhetoric into a success. Systematic reviews are paramount in assessing the current knowledge base; therefore in this review, we sought to synthesis evidence on barriers to achieving PFCC in an ICU setting. Owing to the fact that collaboration is one of the key domains of PFCC, we endeavoured to analyse barriers from perspectives of HCP, family members and patients. In this way, strategies that could foster success of PFCC in ICU can be formulated with prior guidance of the bottlenecks to the concept of PFCC. Specifically, the review sought to:

1. Assess the barriers to achieving PFCC in adult ICUs specifically in the context of HCP, family members and patients.

## 2 METHODS

The Preferred Reporting Items for Systematic Reviews and Meta‐Analysis (Moher, Liberati, Tetzlaff, & Altman, 2009) were applied in this study. This allowed for consideration of different studies while using a systematic approach. When formulating the review question and objectives, we considered the population, intervention, comparison and outcomes (PICO) acronym; however, we acknowledge that this approach is not always fully applicable for qualitative studies.

### 2.1 Search strategy

#### 2.1.1 Scoping search

Initially, a scoping search was done in the Center for Reviews and Dissemination (CRD) database, Cochrane Library, TRIP Database and the Campbell Collaboration databases to ensure that no other review answered the review question or one that was in progress. The review protocol was then registered in the CRD Prospero database (CRD42018086838).

### 2.2 Eligibility criteria and rationale

#### 2.2.1 Types of studies

We included studies if authors reported on barriers to PFCC in adult ICUs. This was done with a specific aim of describing barriers to PFCC in adult ICUs. In addition, we included studies that were published in peer‐reviewed journals in English language from inception to 2018. This timeframe was considered owing to scanty evidence on the research question. We also limited our search to English language due to budgetary constraints. We excluded studies on PFCC that did not report on barriers to PFCC in adult ICUs or those that were conducted in paediatric ICUs or neonatal ICUs (NICU). We also excluded literature reviews; however, these were kept for the discussion section.

#### 2.2.2 Type of participants

Studies on HCP, family members and patients in adult ICUs were included. HCP included either physicians or nurses.

#### 2.2.3 Type of outcome

Studies that reported on barriers to PFCC in adult ICU were included provided they fulfilled the noted criteria above.

#### 2.2.4 Database search

A comprehensive search was carried out to obtain relevant published studies from conception to 2018. The search was carried out in four databases: EMBASE, Cochrane Library, PubMed and Scopus. The final search was done on 23rd January 2018.

#### 2.2.5 Key words

The electronic databases EMBASE and Scopus search were carried out using a combination of the following Medical Subject Headings (MeSH): “ICU,” “family” and “patient‐centered care,” “knowledge,” “barriers,” “nurses,” “physicians” and “adult ICU.” To ensure comprehensive coverage of literature, free title search was also performed using “barriers to PFCC in ICUs”. Furthermore, hand search of reference list of identified papers was done to capture all pertinent materials. Each paper was then assessed whether it met the inclusion criteria.

### 2.3 Data collection process and analysis

#### 2.3.1 Selection of studies

The authors independently inspected the abstract of each identified article and obtained the full text of relevant articles were applicable. The authors independently assessed the articles against the inclusion criteria; they then convened together to discuss and reach consensus on findings where there was doubt about whether the article met the inclusion criteria. We documented the justification for exclusion of studies. We named studies by first author and year of publication (With addition of “a and b” for different studies from the same author and year). Studies were categorized into two categories: The first category included studies that met the inclusion criteria. These were kept in the spreadsheet while the second category included studies that were excluded. These were kept in a different spreadsheet for future reference.

#### 2.3.2 Data‐extraction management

Data were extracted into a piloted data‐extraction spreadsheet. For each study, we recorded the author and year, sample size, sampling method, study population and barriers to PFCC reported. This review used the PICO acronym: The population was HCP, family members and patients in adult ICUs; the intervention was PFCC or in adult ICU while barriers were the outcome/phenomena of interest being assessed across studies.

#### 2.3.3 Data items

The primary outcomes of any study considered were barriers to PFCC or any of the domains of PFCC.

### 2.4 For included studies, we assessed the following

#### 2.4.1 Incomplete outcome data

We recorded the number of participants enrolled in the study, and number of participants evaluated at the end of the study.

##### Summary measures

The principal summary measures were the study setting, samples size, recruitment strategy and barriers to PFCC.

##### Assessment of reporting bias

We constructed a funnel plot to assess the effect of small sample sizes on the main outcomes.

##### Risk of bias in individual studies

Tendencies which precluded consideration of the study objectives in individual studies were blindly assessed by two reviewers. They assessed for and noted where applicable bias during planning, data collection and analysis.

#### 2.4.2 Synthesis of results

Synthesis of aggregate data was done through tabulation of key aspects of the included studies and a narrative description of main findings for each individual study. A data‐driven thematic analysis adopted from Graneheim and Lundman's guidelines (2004) was then undertaken. Following reflection on and abstraction of codes, the three authors subcategorized the data according to the similarities and differences found. Where there was doubt, the authors discussed the categorization and reached consensus. Meta‐analysis was unlikely to be appropriate, due to heterogeneity of study designs included.

#### 2.4.3 Risk of bias across studies

The included studies were of a variety of different study designs so risk of bias was assessed according to generic concepts of selection, performance, attrition and detection of biases appropriate to each study design. Selection bias was assessed according to the sampling method. Critical appraisal and methodological rigour were observed in a collaborative way among the authors to ensure that pertinent publications were not missed. Despite the heterogeneity of the study population, we did not develop appropriate subgroups after the scanning of the full text of selected studies.

#### 2.4.4 Ethical considerations

No ethical clearance was required for this study.

## 3 RESULTS

**Figure 1:**
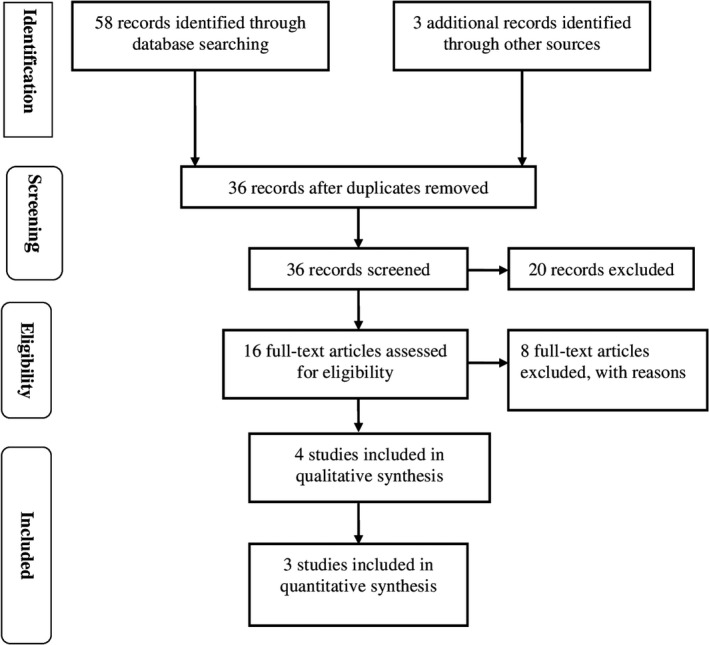
Flow diagram of study selection

Database search conducted using the keywords described in the previous section resulted in 58 articles initially (Figure 1). Further hand search using the reference list yielded no articles. The foremost rationale for exclusion in the screening stage was related to purpose of the review and other interventions other than PFCC. With regard to excluded articles, two articles were systematic reviews; six articles were carried out in NICU; 37 articles reported on the outcomes that were not related to the review question while three articles were expert opinions. Seven articles met the inclusion criteria and were included in the review (Table 1). Of the seven articles, three were quantitative and four were qualitative (Figure 1). With respect to data collection approaches, three of the four qualitative studies used semi‐structured interviews (Esmaeili, Cheraghi, & Salsali, 2014; Moore et al., 2017; Tunlind, Granström, & Engström, 2015) while the other used focused group meetings (Riley et al., 2014). With regard to country of origin, two articles were conducted in the USA, two from Sweden and one article each from, the Netherlands, Israel and Iran (Table 1).

**Table 1:** Study design and methods of included studies

Author (year) country	Aim	Participants	Study design and methods	Findings
Tunlind et al. (2015) Sweden	To describe critical care nurses’ experience of performing nursing care in a highly technological care environment	8 critical care nurses	Qualitative: Personal semi‐structured interviews using an interview guide with reference to Graneheim and Lundman (2004) Analysed using content analysis of thematic transcription	Technology is a barrier to patient‐centred care
Esmaeili et al. (2014) Iran	To explore nurses’ attitude and experience towards the barriers to achieving patient‐centred care in the critical care setting	21 nurses working in intensive care units in teaching hospitals in Iran	Qualitative: In‐depth semi‐structured interviews Analysed by thematic analysis approach with reference to Braun and Clarke (2006)	Three themes: (a) lack of common understanding of teamwork, (b) individual barriers and (c) organizational barriers
Riley et al. (2014) USA	To understand perceptions on patient‐centred ICUs among patients’ family members, physicians and nurses	8 family members, 3 physicians and 7 nurses	Qualitative: Focused group meetings Analysed by transcription from voice‐recorded tapes	Competing roles of control over patient care
van Mol et al. (2017) the Netherlands	To evaluate the impact of multiple supportive interventions perceived by patients’ relatives and HCP in ICU	334 relatives and unspecified number of HCP	Quantitative: time trend survey using questionnaires	Time constraints, daily rushed workloads and competing priorities
Ganz and Yoffe (2012) Israel	To determine the attitude of nurses towards PFCC and family presence during resuscitation and its association with PFCC	96 ICU and coronary care unit nurses	Quantitative: 5 questionnaires used to assess outcome variables	Time constraints, difficult patients, conflict with medical doctors, discouraged by medical doctors to discuss some issues with family, lack of support from fellow nurses, outside nursing practice, visiting policy, no family visitations, communication barriers between experienced doctors and nurses, angry family, unrealistic family expectations, personal difficulty in dealing with the family, lack of private space for family and language difficulty
Downey et al. (2006) USA	To evaluate 3 questionnaires measuring nurses perspectives on family‐centred end of life care in intensive care units and to show the usefulness of the questionnaires	141 critical care nurses	Quantitative: using questionnaires	Patient/family‐related barriers and system/team‐related barriers
Moore et al. (2017) Sweden	To explore barriers and facilitators to the delivery of person‐centred care interventions in different healthcare settings	18 Researchers across healthcare settings	Qualitative: Interviews using semi‐structure guide Analysed transcriptions with reference to grounded theory	Traditional practices and work environments built on the biomedical model, HCP's attitudes and issues in development of PCC interventions

Note

HCP: healthcare providers; ICUs: intensive care units.

This review represents analysis of 618 participants. Of these, 342 were family members and 273 were nurses, three physicians and 18 were researchers from different healthcare settings. The study of van Mol contributed more than half of the study population with 334 participants (Table 1). Most studies (*N* = 5) focused on entirely assessing barriers related to delivering PFCC (Downey, Engelberg, Shannon, & Curtis, 2006; Esmaeili et al., 2014; Ganz & Yoffe, 2012; Moore et al., 2017; Riley et al., 2014) and two studies indirectly assessed barriers to PFCC (van Mol et al., 2017; Tunlind et al., 2015). The four included qualitative studies are comprised of one grounded theory and two descriptive studies. The synthesis approach of qualitative research was done using the Qualitative Assessment and Review Instrument tool from the Joanna Briggs Institute. This also provided a structured process for extracting and aggregating findings in relation to the purpose of the review. The findings of this review described barriers to PFCC in adult ICUs using four categories: The first category of barrier is lack of understanding of PFCC, the second category of barrier falls under organizational barriers, the third category falls under individual barriers and the fourth category falls under interprofessional barriers. For an overview, see Table 2.

**Table 2:** Subcategories of barriers to patient and family‐centred care (PFCC)

Subcategories	Main categories
Lack of common understanding of teamwork	Lack of understanding of PFCC
Competing roles of control over patient care	
Lack of support from fellow nurses	
No family visitations	
Unrealistic family expectations	
Issues in development of PCC interventions	
Daily rushed workloads	Organizational‐related barriers
Visiting policy	
Lack of private space for family	
Time constraints	Individual‐related barriers
Competing priorities	
Difficult patients	
Angry family	
Technology is a barrier to patient‐centred care	
Healthcare provider's attitudes	
Personal difficulty in dealing with the family	
Language difficulty	
Conflict with medical doctors	Interdisciplinary barriers
Discouraged by medical doctors to discuss some issues with family	
Communication barriers between experienced doctors and nurses	
Traditional practices and work environments built on the biomedical model	

### 3.1 Lack of understanding of what needs to be done to achieve PFCC

This theme was abstracted from the following subtheme: lack of support from fellow nurses, unrealistic expectations, no family visitations (Ganz & Yoffe, 2012), competing roles of control over patient care (Riley et al., 2014) and issues in development of Patient‐Centered Care (PCC) interventions (Moore et al., 2017) (Table 2).

### 3.2 Organizational‐related barriers

This category was abstracted from the following main categories: inappropriate environment to foster PFCC, lack of guidelines on PFCC and lack of role models at workplaces to champion PFCC (Esmaeili et al., 2014). Inappropriate work environments have been cited as: nurse shortages, no support to HCP in achieving PFCC, workload, high nurse–patient ration, overcrowded hospitals, burnout of HCP and lack of reinforcement of positive PFCC behaviours and poor ICU design. Guidelines and policies that foster PFCC in ICU span from lack of defined guidelines and tools to provide PFCC to lack of communication policies that foster family and HCP communication from time of admission to discharge. With regard to lack of model co‐workers in the ICU, studies have cited that oftentimes HCP argued that PFCC is only talked about without doing anything and managers failed to provide a suitable environment to provide PFCC.

### 3.3 Individual barriers

Individual barriers cited across studies include the following: lack of motivation, lack of holistic view of care and lack of time. Lack of motivation was described in different versions across studies. Esmaieli's study noted that nurses reported the following which reflect lack of motivation, they included the following: lack of interest, limited beliefs in PFCC, poor motivation by colleagues, low nurse income, job satisfaction, trying our level best despite organizational problems (Esmaeili et al., 2014). Defining characteristics reported with regard to lack of holistic view of care includes HCP not giving attention to all patients’ and family needs from admission to discharge. In addition, lack of time has been noted across different studies inform of reports of work overload, and lack of time to explain all information to patients’ families.

### 3.4 Interprofessional‐related barriers

Various interdisciplinary barriers to PFCC were reported across the studies. These have been reported in different forms which include the following: tension between HCP's professional responsibilities to discuss likely patient functional outcomes versus uncertainty about their ability to predict such outcomes. Others included unrealistic optimistic expectations of recovery among ICU surrogates, minimal confidence applying existing outcomes of research to individual patients and unrealistic expectations about operational capabilities (Downey et al., 2006; Ganz & Yoffe, 2012; Tunlind et al., 2015; Turnbull et al., 2016).

## 4 DISCUSSION

The pivotal objective of this review was to assess the barriers to achieving the concepts of PFCC specifically those encountered in adult ICUs. Kirkevold (1997) argued that integrating findings from empirical studies is a method of building in‐depth understanding of a given phenomenon which enhances knowledge building. Noteworthy, few studies have reported on the barriers to its implementation in the adult ICUs. Indeed, we did not find any study assessing barriers to PFCC in Africa specifically in intensive settings. This has been reported elsewhere in 2016 review that sought to assess patient and family involvement in adult critical care and intensive care settings. Therefore, we recommend more empirical studies assessing the bottlenecks to PFCC especially in the intensive care settings.

Barriers to PFCC in the intensive care setting reported in this review fit well into the four categorizations, that is, lack of understanding of what needs to be done to achieve PFCC, organizational, individual and interprofessional barriers. We considered this classification to facilitate a systems understanding of barriers to PFCC. We also hope that this categorization may be helpful in recommending targeted interventions to a specific faction of the system in attempt to implement PFCC. This has been done elsewhere, for instance in Esmaeili's (2014) study, they categorized their findings into three themes, that is, lack of understanding of teamwork, individual barriers and organizational barriers. Our categorization is quite similar to that reported in Esmaeili's (2014) study with further expansion of a fourth categorization. Indeed, our findings support selective or constructive measures for system level PFCC interventions.

Regarding quantitative studies, different tools have been used across studies. Questionnaires used in quantitative studies identified in this review include the Barriers to Providing Family‐Centered Care‐Revised (Downey et al., 2006; Ganz & Yoffe, 2012). The Barriers to Providing Family‐Center Care‐Revised is a 10‐item scale designed to measure 10 potential barriers to providing PFCC grouped into patient/family barriers and system/team barriers (Ganz & Yoffe, 2012). The questionnaire asks the respondent whether the barriers exist by giving a yes/no answer. Findings of this review reveal that on top of the items assessed in this questionnaire other items such as questions to reflect motivation of HCP, holistic view of both the family/patient and HCP to care, presence of guidelines/policy on PFCC and role models in the ICU in the context of achieving PFCC should be added to such tools. This could to cover a wider picture of the barriers. Other quantitative studies used questionnaires developed from literature while data collection in qualitative studies used interviews. These mainly used open‐ended questions to elicit barriers to PFCC. This method is effective for eliciting responses on themes that are later organizing and analysing the data which enriches the outcomes. Furthermore, critics to systematic reviews have noted the significant exclusion of findings from qualitative studies (Dixon‐Woods, Agarwal, Jones, Young, & Sutton, 2005); for this reason, we decided to explore findings from both qualitative and quantitative research. This even allowed a wider search for studies owing to the fact that studies assessing barriers to PFCC in critical care settings are scanty. Thus, to keep in line with our objective, we synthesized findings from different qualitative and quantitative research traditions into “qualitized” categories. We found this approach to be appropriate owing to the fact that we were not interested in how the barriers were quantified but rather in giving a thorough prescription of barriers to PFCC in intensive care settings. Inclusion of studies was mainly based on critical appraisal of each study against the inclusion criteria and peer‐reviewing by the authors. It has been reported elsewhere that when including studies with different designs, it is paramount to evaluate the quality of each study where outliers are suspected (Whittemore & Knafl, 2005). Albeit that sentiment, our findings fit well into the final categorizations. Another methodological issue in our study was that one can assume that van Mol's (2017) study reported findings within‐ and between‐supportive interventions perceived by patients’ relatives and HCP in ICU; however, some findings were presented closely to barriers to PFCC in ICU.

Positive outcomes of patient and family‐centred care cannot be achieved in isolation. In fact, collaboration forms on one of the core concepts of PFCC (Tzelepis et al., 2014). Lack of understanding of what is need to achieve PFCC is commonly cited as, lack of teamwork. Teamwork helps to achieve some of the components of PFCC, that is, care coordination and integration. This barrier can be used to inform the HCP in ICUs of the need for teamwork so as to achieve PFCC. Care coordination has been identified as the key strategy to provide high‐quality health care through assuring timely access to resources needed to optimize health care services (Tzelepis et al., 2014).

In addition to focusing on patient engagement, focus should be broadened to include relationships among ICU staff. If the staffs are not working together effectively, this may interfere with establishment of positive staff–patient relationship. Care integration involves interface among different HCP and systems to offer comprehensive services, especially for patients with multiple healthcare needs (Verma & Navarro, 2015). In addition, owing to the diverse needs of the patient and family in the ICU, cooperation between all members of the ICU healthcare team and the family is paramount for better outcomes. Uncoordinated teams may lead to mismatch in communication, delayed information to the family and delayed discharge. The notion that some players of the healthcare team lack time to interact with the patients’ family may be attributed to lack of teamwork among the healthcare team. Unsuccessful collaboration could lead to failure to achieving PFCC in the ICU. It has been associated with poor patient and patients’ family satisfaction, low nurse retention, suboptimal patient outcomes and safety, communication problems, poor understanding of other teams’ working conditions and environment (Downey et al., 2006; Esmaeili et al., 2014).

Indeed, studies have reported that lack of coordination may lead to duplication of work between ICU and other departments such as radiology and laboratory which lead to reduction in quality of care. Interventions aimed at assigning and distribution of roles among the healthcare team member and an assigned family member to the healthcare team could ensure effective team coordination. Creating well functioning and effective interdisciplinary teams is particularly important in high‐intensity care areas such as the ICU. Potential strategies include the following: team building activities in ICU, change in attitude from an “I to us” mentality.

To be truly patient and family‐centred, organizations must support the concept of PFCC through creating a conducive environment for the staff, patient and the patient's family. Several barriers related to the working environment have been tied to inappropriate environment to foster PFCC including lack of guidelines on PFCC and lack role models at workplaces to champion PFCC. Other barriers include nurse shortages, no support to HCP in achieving PFCC, workload, high nurse–patient ration, overcrowded hospitals, burnout, lack of reinforcement of positive PFCC behaviours and poor ICU design (Esmaeili et al., 2014; Riley et al., 2014). Such organizational barriers could hinder full realization of PFCC. Similar findings have been reported by Luxford, Safran, and Delbanco (2011) and those from the widely publicized IOM reports which included issues related to organizational structure, incentives, team conflicts related to trust issues, mutual respect and roles (Ganz & Yoffe, 2012). For patient‐centred care to become truly embedded in an organization and in the broader healthcare system, it must be dependent on reliable systems, rather than the behaviour of individuals. Eisenberg alleged that “medicine is social in nature and this is more applicable inside a hospital.” Organizational and teamwork factors have profound impact on quality and care outcomes, particularly in the ICU where organizational and teamwork factors are central to daily operations (Moeckli, Cram, Cunningham, & reisinger, 2013; Weled et al., 2015; Young, Chan, & Cram, 2011).

Guidelines and policies that foster PFCC in ICU span from lack of defined guidelines and tools to provide PFCC to lack of communication policies that foster family and HCP communication from time of admission to discharge. Systems must be designed to deliver consistent, patient‐centred results, and there should be guidelines in place to hold accountable those organizations that fail to design such systems. With regard to lack of model co‐workers in the ICU, studies have cited that oftentimes HCP argued that PFCC is only talked about without doing anything and managers failed to provide a suitable environment to provide PFCC. Organizations need to consider PFCC as a partnership with all stakeholders including the HCP. Luxford's study on promoting patient‐centred care showed nine key organizational attributes and processes derived from interviews with key hospital managers for successful PFCC which include strong, vomited senior leadership, effectively communicated vision, active patient and family engagement throughout the institution, enduring focus on satisfying staff, active quantification and feedback from patients, adequate resourcing of care delivery redesign, staff capacity building, accountability and incentives, instilling a culture that is positive to change and learning (Luxford et al., 2011).

Our review identified individual‐related barriers; we also highlight potential areas for improvement based on the findings from previous studies. Individual‐related barriers cited across studies are numerous, these included lack of motivation, lack of holistic view of care and lack of time. Lack of motivation was described in different versions across studies. Potential strategies to individual barriers could include having clearly defined roles and responsibilities related to PFCC dimensions.

Esmaeili et al. (2014) argued that prepared, engaged HCP are a fundamental precursor to achievement of PFCC. Indeed, if HCP lack interprofessional collaboration; the clarity and accuracy of the communication are compromised (Dalal, Bates, & Collins, 2017). We also highlight that optimistic expectations for recovery among ICU surrogates and minimal confidence applying existing evidence to individual patients could hinder some aspects of PFCC such as shared decision‐making and communication on prognosis. To realize the benefits and implementation of PFCC, potential solutions include awareness of facility resources, clearly defined expectations, role reversal and quality improvement feedback mechanisms. Furthermore, this could foster sharing ideas, barriers and achievements.

Conclusively, training HCP on the relevance of PFCC might lead to stronger awareness, benefits and implementation of PFCC. In addition, the combined knowledge and skills from collaboration between HCP and the patients’ family need to be appreciated, acknowledged and embraced.

### 4.1 Limitations of the study

The studies identified in this review for analysis are mainly from developed countries with only one article from a middle‐income country; as such generalizing the barriers identified to low‐income economies is questionable. We recommend more studies in low‐ and middle‐income countries. Another limitation of this review is that few studies were included in the review.

## 5 CONCLUSIONS

This review presents barriers to PFCC in an intensive care environment. The findings challenge organizational and HCP commitment, teamwork, an environment that fosters PFCC, effective communication and knowledge on PFCC. Knowledge on barriers that hinder a patient and family‐centred environment can help in identification of solutions and improved patient and family satisfaction. The results also challenge the belief that PFCC can be achieved individually but rather on a system‐based approach. Insights into these barriers can guide interventions aimed at implementing or improving PFCC in adult ICUs. Barriers to PFCC in ICU are more or less similar to those in other settings. This evidence can be used to develop further research.

## CONFLICT OF INTEREST

The authors declare no conflict of interest.
